# Evaluation of hard palate bone thickness on cone -beam computed tomographs for orthodontic mini-implant placement: A cross-sectional study

**DOI:** 10.4317/jced.63661

**Published:** 2026-02-26

**Authors:** Silvia Huaynate-Cuadrado, Manuel Gustavo Chávez-Sevillano, Daniel Blanco-Victorio, Sandra P. Palomino-Gomez, Jessica Arieta-Miranda, Catia Cardoso Abdo Quintão, Luis Fernando Pérez-Vargas

**Affiliations:** 1 Faculty of Dentistry, National University of San Marcos, Lima, Perú; 2 Faculty of Science and Engineering,Cayetano Heredia Peruvian University, Lima, Perú; 3 Faculty of Dentistry, Rio de Janeiro State University, Rio de Janeiro, Brasil

## Abstract

**Background:**

This study aimed to determine the bone thickness of the hard palate using cone-beam computed tomography (CBCT) to identify suitable sites for orthodontic mini-implant placement.

**Material and Methods:**

Fifty CBCT scans were analyzed, comprising 31 women (mean age, 25.77 ± 7.16 yr.) and 19 men (mean age, 26.16 ± 6.92 yr.), aged 18-45 yr. Measurements were obtained using Real Scan 2.0 software on coronal sections at 4, 8, 12, and 16 mm posterior to the incisal foramen (IF) and at 0, 3, and 6 mm lateral to the midsagittal plane (MSP) on both sides. Statistical analysis was performed in Stata v15 using the Shapiro--Wilk, Mann--Whitney U, Wilcoxon, Kruskal--Wallis, and Dunn's post hoc tests, with a 95% confidence level.

**Results:**

No significant differences were observed between the right and left sides. The greatest mean bone thickness was found at slice 4 along the MSP (5.91 ± 1.72 mm). A significant decrease in bone thickness was noted toward the lateral and posterior regions, except at slice 16. Men exhibited significantly greater bone thickness than women at 4, 8, and 12 mm. Slice 4 corresponded to the region between the first and second premolars in 48% of participants, whereas slice 8 corresponded to the level of the second premolar in 76% of participants.

**Conclusions:**

The region of the hard palate with the greatest bone thickness was identified at slice 4 (4 mm posterior to the IF) at 0, 3, and 6 mm lateral to the MSP, located in 48% of subjects between the first and second premolars. Men demonstrated significantly greater bone thickness than women at all slices except slice 16.

## Introduction

One of the primary concerns among orthodontic patients is the overall treatment duration. This period has been shortened through the use of novel devices and techniques, such as orthodontic miniscrews (1). Miniscrews, also referred to as temporary anchorage devices, can be placed in various maxillary and mandibular regions, including the hard palate, the infrazygomatic crest, and the mandibular buccal shelf (2). Cone-beam computed tomography (CBCT) has become an essential tool in orthodontics because it provides high-resolution, three-dimensional images of dental and maxillofacial structures across multiple planes (1). Unlike traditional radiographs such as lateral cephalometric and panoramic images, CBCT offers minimal distortion and allows for precise, individualized measurements (3). The hard palate is considered a favorable site for miniscrew placement due to its keratinized mucosa (4), cortical bone thickness exceeding 1 mm (2 , 5), and limited presence of anatomical structures prone to complications, such as inflammation (4) or nasal cavity perforation (6). Additionally, this site offers good long-term stability (7) and is generally well accepted by patients (8). Owing to these advantages, miniscrews have been successfully used in several orthodontic procedures, including maxillary expansion, molar and incisor intrusion, molar distalization and mesialization, incisor retraction, maxillary protraction, and canine traction (9 , 10). Previous studies have reported variable results regarding palatal bone thickness. Gracco et al. (11) and Winsauer et al. (12) found bone thicknesses greater than 8 mm at the mid-palatal suture with no significant sex-related differences. In contrast, Poorsattar--Bejeh et al. (13) reported average values exceeding 6.9 mm and noted significant differences between men and women, as well as among populations from different regions. Similarly, Vidalon et al. (14) observed mean thicknesses of 12.21 mm at 4 mm posterior to the incisal foramen (IF). Given the variability in the literature and the clinical importance of selecting optimal palatal sites for miniscrew placement, the present study aimed to evaluate hard palate bone thickness using CBCT as a reference for orthodontic mini-implant insertion.

## Material and Methods

**Figure 1:**
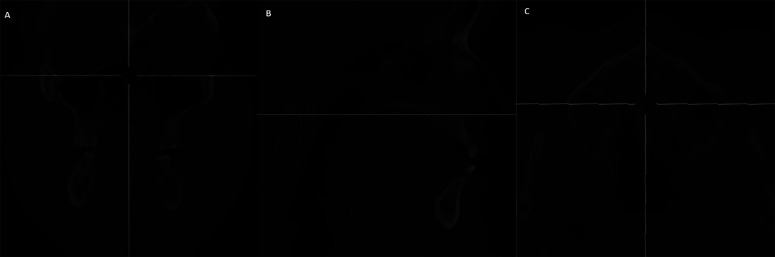
Standardization of CBCT image positioning: (A) Coronal view, (B) Sagittal view, and (C) Axial view.

This study was approved by the Ethics Committee of the Faculty of Medicine, National University of San Marcos (Protocol No. 0333). CBCT images were obtained from the Radiology Service database of the School of Dentistry, National University of San Marcos (UNMSM.). The sample size was estimated statistically considering a confidence level of 95% and a 5% margin of error. Due to difficulties in obtaining records of patients with specific characteristics for the study, a total of 50 CBCT images were selected using convenience sampling. The sample comprised 31 women (mean age, 25.77 ± 7.16 yr) and 19 men (mean age, 26.16 ± 6.92 yr), aged 18 to -45 years. Inclusion criteria were the presence of a complete upper dentition, with or without third molars, and high-quality CBCT images. Exclusion criteria included the presence of supernumerary teeth, impacted teeth affecting the palatal region, and any history of orthodontic or orthognathic treatment. The CBCT scans were acquired using exposure parameters of 90 kVp and 5 mA, image dimensions of 640 x× 640 x× 512 mm, and a voxel resolution of 0.303 x× 0.0303 x303 × 0.0303 mm. Images were analyzed using Real Scan 2.0 software (PointNix Co. Ltd., Seoul, Korea). All digital imaging and communications in medicine images were standardized, and each tomographic volume was positioned in the Multiplanar Reconstruction (MPR) mode. In the coronal view, the orbital plane was oriented parallel to the horizontal reference line and perpendicular to the midsagittal plane (MSP). In the sagittal view, the Frankfort plane (FP) was aligned horizontally. The MSP was defined as the line passing through the anterior nasal spine (ANS) and posterior nasal spine (PNS), following the methodology of Poorsattar--Bejeh et al. (13) (Fig. 1).

1

**Figure 2:**
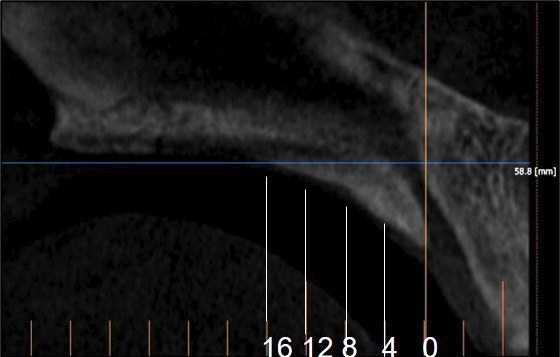
Sagittal view showing slices obtained at 0, 4, 8, 12, and 16 mm from the posterior border of the incisive foramen (IF).

The FP was vertically displaced at the ANS level for measurement alignment. In the axial view, the cross -sectional tool was used to trace a line from the ANS to the PNS. Coronal slices were then generated along the hard palate with a slice thickness of 1 mm and a 4 -mm interslice distance. These slices were designated as slices 0, 4, 8, 12, and 16. Slice 0 corresponded to the distal margin of the IF (Fig. 2).

2

**Figure 3:**
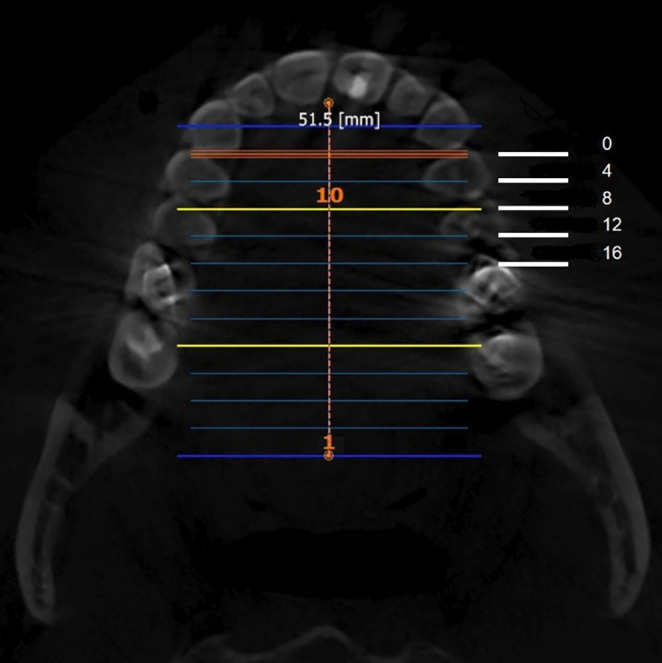
Axial view of the palate illustrating measurements taken at 0, 4, 8, 12, and 16 mm posterior to the IF and their relationship to the teeth.

The anatomical location of each slice was identified and recorded relative to the upper dentition: -canine, first premolar, second premolar, and first molar (Fig. 3).

3

**Figure 4:**
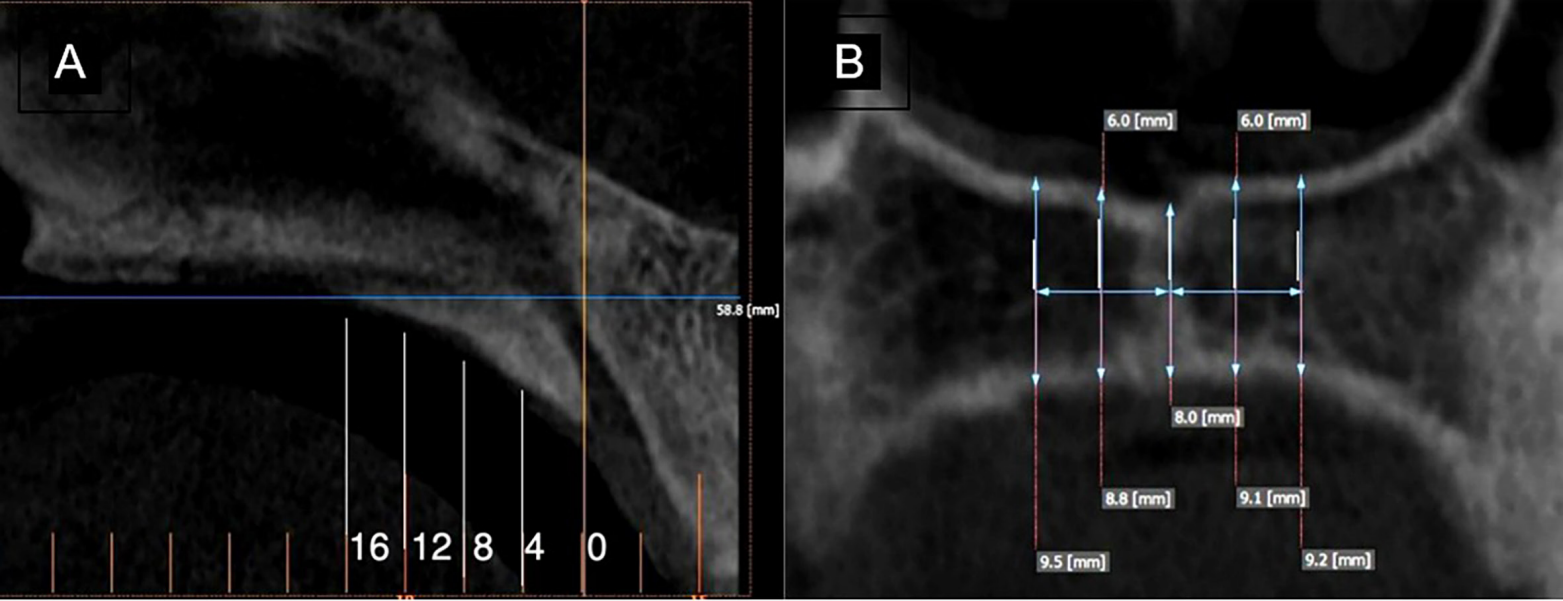
Representative sections of the palate: (A) Sagittal slices at 0, 4, 8, 12, and 16 mm from the IF and (B) Coronal slices at 0, 3, and 6 mm from the transverse dental crest (TDC.)

Bone thickness measurements were obtained following the methodology described by Gracco et al. (11) and subsequent studies (5 , 13 , 15 , 16). Measurements were performed in the coronal view, perpendicular to the horizontal plane, extending from the lower cortical plate of the hard palate to the upper cortical plate of the nasal floor. Measurements were taken at the MSP and at 3 and 6 mm lateral to both right and left sides in slices 4, 8, 12, and 16, resulting in 20 measurements per CBCT scan (Fig. 4).

4

- Statistical Analysis Data analysis was performed using Stata version 15 (StataCorp LLC, College Station, TX, USA). Based on prior studies (4 , 13 , 15), 100 measurements (right and left sides combined) were evaluated from 50 CBCT scans. To assess intra-examiner reliability, 20 CBCT scans were randomly selected, and measurements were repeated after 15 days by the principal investigator (SH). The intraclass correlation coefficient (ICC) was calculated to evaluate measurement consistency. The Shapiro--Wilk test was used to assess normality; as the data were not normally distributed, non-parametric tests were applied. The Wilcoxon signed-rank test was used to compare the right and left sides, and the Mann--Whitney U test was applied to compare male and female subjects. Differences among the various coronal sections were analyzed using the Kruskal--Wallis test followed by Dunn's post hoc test. Statistical significance was set at p &lt; 0.05.

## Results

Assessment of intra-observer reliability revealed a high ICC of 0.978, indicating excellent measurement consistency. A total of 100 bone thickness measurements were obtained from 50 CBCT scans. The greatest mean bone thickness was observed at slice 4 (5.91 ± 1.76 mm at the MSP), whereas the lowest mean value was recorded at slice 16, 6 mm lateral to the MSP (1.84 ± 1.03 mm.). A progressive decrease in bone thickness was observed as both the anteroposterior distance from the IF and the lateral distance from the MSP increased (Table 1).

Table 1

When comparing bone thickness between sexes, statistically significant differences (p &lt; 0.05) were found at 0, 3, and 6 mm from the MSP and at 4, 8, and 12 mm posterior to the IF. No significant differences were noted at 16 mm from the IF. Among men, the highest mean bone thickness (7.06 ± 1.75 mm) was recorded at slice 4, with a gradual decrease as the distance from the IF and MSP increased, except at slice 4 where value remained consistent. In women, the highest mean thickness was 5.23 ± 1.89 mm, observed at slices 4 and 16, 6 mm lateral to the MSP (Table 2).

Table 2

In both the male and female groups, statistically significant differences were observed in the anteroposterior direction (slices 4, 8, 12, and 16 mm) at 0, 3, and 6 mm lateral to the MSP, except at slices 12 and 16 (p &gt; 0.05; Table 3).

Table 3

Similarly, significant differences were found in the lateral direction (0, 3, and 6 mm from the MSP), except at slice 4 (p &gt; 0.05; Table 4).

Table 4

The positional distribution of the coronal slices relative to the maxillary dentition showed that slice 4 (4 mm posterior to the IF) corresponded to the region between the first and second premolars in 48% of cases. Slice 8 (8 mm posterior to the IF) was located at the level of the second premolar in 76% of cases; slice 12 (12 mm posterior to the IF) corresponded to the area between the second premolar and first molar in 46% of cases; and slice 16 (16 mm posterior to the IF) was located at the level of the first molar in 94% of cases.

## Discussion

All CBCT scans evaluated in this study included subjects with complete upper dentition and no palatal abnormalities. This inclusion criterion limited the expansion of the sample size. However, the final sample size was comparable to those used in previous studies by Poorsattar--Bejeh et al. (13), Kang et al. (4), and Bonangi et al. (15). Because the sample consisted exclusively of adults, age and growth were not considered variables in the analysis (4 , 11 , 13 , 17 - 19). An ICC of &gt;0.9 indicated excellent intra-observer reproducibility, consistent with findings from previous research (11). No statistically significant differences were observed between the right and left sides of the palate, in agreement with the results of Holm et al. (19), Hourfar et al. (20), Poorsattar-Bejeh et al. (13), Ryu et al. (21), Gracco et al. (11), and Garleitner et al. (22). This symmetry may be attributed to the exclusion of subjects with facial asymmetries or skeletal dimorphisms. Several authors, including Gracco et al. (11), Poorsattar-Bejeh et al. (13), Winsauer et al. (12), and Bonangi et al. (15), have reported that the region approximately 4 mm posterior to the IF exhibits greatest bone thickness. The present study partially supports these findings, with the highest mean values recorded at 3 and 6 mm lateral to the MSP in slice 4 (Table 1). These results indicate that this region is suitable for orthodontic miniscrew placement. Similarly, Zhu et al. (23) identified the optimal region between the first and second premolars, emphasizing that bone density and mucosal thickness should also be considered during clinical planning. The greatest mean value in the present study (5.91 ± 1.72) was lower than that reported by Gracco et al. (11) (10.35 ± 3.24 mm) and Poorsattar-Bejeh et al. (13) (6.96 ± 1.81 mm). These discrepancies may be attributed to differences in population ethnicity and sample origin, as well as variations in measurement methodology. Taghizadeh (24) demonstrated that differences of 0.18 to -3.69 mm can occur depending on the reference plane used (IF-PNS vs. ANS-PNS), with higher values typically associated with the IF-PNS plane. Regarding sex differences, this study found significantly greater bone thickness in men than in women. These findings contrast with those of Gracco et al. (11) and Summer (17), who reported no significant differences between sexes, but align with studies by Kang et al. (4), Holm et al. (19), Taghizadeh (24), and Gholinia et al. (25), all of which documented thicker palatal bone in male subjects. Because the sagittal slice at zero position was located along the posterior border of the IF-an anatomically sensitive region for miniscrew insertion-palatal thickness at this site was excluded from clinical consideration. In terms of clinical correspondence, slice 4 was positioned between the first and second premolars in 48% of cases, consistent with the findings of Kim et al. (26). However, this differs from Hourfar et al. (20), who located slice 4 between the canine and the first premolar, and Winsauer et al. (12), who placed it at the level of the first premolar cusps. The posterior border of the IF coincided with the clinical location of the first premolar in 50% of cases, highlighting the importance of avoiding injury to the nasopalatine neurovascular bundle. Nevertheless, Henriksen et al. (27) and Bernhart et al. (6) demonstrated that the nasopalatine canal typically does not extend more than 3 mm from the MSP, thereby reducing the likelihood of perforation when proper care is taken. Negrisoli et al. (28) and Gibas-Stanek et al. (29) suggested that the anterior region near the MSP is the most favorable site for miniscrew-assisted rapid palatal expander (MARPE) placement. However, the present study found considerable variability in bone thickness, reinforcing the need for individualized CBCT-based assessment before selecting the miniscrew insertion site and length. The significant variability observed in both sagittal and transverse measurements (except in slice 4; Tables 3 and 4) underscores the necessity of larger sample sizes in future research. Moreover, as emphasized by Lyu et al. (30) and Wang et al. (31), inter-population anatomical differences should be considered when analyzing palatal bone morphology, as genetic and racial factors may influence bone characteristics.

## Conclusions

The area of the hard palate with the greatest bone thickness was observed in slice 4 at 0, 3, and 6 mm on both the right and left sides of the MSP, corresponding in 48% of the samples to the region between the first and second premolars. Men exhibited significantly greater palatal bone thickness than women in all slices, except in slice 16. The considerable variability observed in bone thickness emphasizes the importance of individualized assessment using CBCT prior to selecting the miniscrew insertion site.

## Figures and Tables

**Table 1:** Bone thickness of the hard palate at 4, 8, 12, and 16 mm from the IF to 0, 3, and 6 mm lateral to the MSP.

Coronal position	Lateral position	Mean	SD	p50	Interquartile range IQR	Min	Max
Section 4	At level of MSP	5.91	1.72	5.7	2.8	2.9	9.4
	3 mm from MSP	5.71	1.89	5.35	2.9	2.1	9.8
	6 mm from MSP	5.88	2.14	5.9	3.1	2	11.1
Section8	At level of MSP	4.54	1.49	4.5	2.1	1.9	8.5
	3 mm from MSP	3.82	1.48	3.6	2.5	1.4	7.1
	6 mm from MSP	3.10	1.41	2.95	2.4	0.8	6.6
Section12	At level of MSP	3.89	1.25	3.95	2	1.7	6.8
	3 mm from MSP	3.04	1.24	2.95	1.9	1	5.8
	6 mm from MSP	2.11	1.06	1.7	1.5	0.7	5.2
Section16	At level of MSP	3.69	1.11	3.7	1.8	1.8	6.1
	3 mm from MSP	2.89	1.09	2.95	1.6	0.9	5.3
	6 mm from MSP	1.84	1.03	1.5	1.6	0.6	4.7

*MSP = Mid sagital plane

**Table 2:** Bone thickness of the hard palate in the coronal plane at 4, 8, 12, and 16 mm from the IF at 0, 3, and 6 mm lateral to the MSP in male and female patients.

Coronal position	Lateral position	Gender						
		Male			Female			
		Mean	SD	p50	Mean	SD	p50	p*
Section4	At level of MSP	7.06	1.75	7.40	5.20	1.36	4.80	0.001
	3 mm from MSP	6.86	2.06	7.25	5.00	1.41	4.75	0.000
	6 mm from MSP	6.94	2.15	7.30	5.23	1.89	4.95	0.000
Section8	At level of MSP	5.39	1.57	5.60	4.02	1.18	3.80	0.001
	3 mm from MSP	4.80	1.43	5.05	3.22	1.20	3.00	0.000
	6 mm from MSP	3.87	1.34	4.15	2.62	1.25	2.35	0.000
Section12	At level of MSP	4.61	1.31	4.80	3.44	0.99	3.40	0.001
	3 mm from MSP	3.60	1.28	3.90	2.70	1.10	2.50	0.001
	6 mm from MSP	2.47	1.13	2.45	1.88	0.97	1.60	0.006
Section16	At level of MSP	3.70	1.13	3.70	3.68	1.12	4.80	0.992
	3 mm from MSP	3.10	1.14	3.10	2.75	1.06	4.75	0.131
	6 mm from MSP	1.94	1.01	1.60	1.78	1.04	4.95	0.288

* Mann-Whitney U test for comparison between female and male, p<0.05 significant

**Table 3:** Comparison of hard palate bone thickness in the anteroposterior direction at 0, 3, and 6 mm lateral to the MSP in male and female patients.

Coronal position	Lateral position	Male			Female			Total		
		Mean	p50**	p*	Mean	p50**	p*	Mean	p50**	p*
0 mm from MSP	Section4	7.06	7.40ab	0.01	5.20	4.80ª	0.01	5.91	5.70ab	0.01
	Section8	5.39	5.60ab		4.03	3.80ab		4.55	4.50ab	
	Section12	4.62	4.80b		3.45	3.40b		3.89	3.95b	
	Section16	3.71	3.70bc		3.68	3.80c		3.69	3.70bc	
3 mm from MSP	Section4	6.86	7.25a	0.01	5.01	4.75ª	0.01	5.71	5.35a	0.01
	Section8	4.80	5.05a		3.23	3.00ab		3.83	3.60a	
	Section12	3.60	3.90a		2.71	2.50b		3.05	2.95a	
	Section16	3.11	3.10c		2.76	2.70c		2.89	2.95c	
6 mm from MSP	Section4	6.95	7.30a	0.01	5.24	4.95a	0.01	5.89	5.90a	0.01
	Section8	3.88	4.15a		2.62	2.35a		3.10	2.95a	
	Section12	2.48	2.45a		1.88	1.60a		2.11	1.70a	
	Section16	1.94	1.60c		1.78	1.40c		1.84	1.50c	

* Kruskal-Wallis test for comparison of thicknesses in the sagittal (anteroposterior) direction, p<0.05 significant* Dunn’s Post-Hoc test for comparison of thicknesses in the sagittal (anteroposterior) direction: equal letters, significant difference, p<0.05

**Table 4:** Comparison of hard palate bone thickness in the transverse direction at 0, 3, and 6 mm lateral to the MSP in male and female patients.

Coronal position	Lateral position	Male			Female			Total		
		Mean	p50**	p*	Mean	p50**	p*	Mean	p50**	p*
Cut 4	At level of MSP	7.06	7.40a	0.90	5.20	4.80a	0.71	5.91	5.7ª	0.73
	3 mm from MSP	6.86	7.25ab		5.01	4.75ab		5.71	5.35ab	
	6 mm from MSP	6.95	7.30ac		5.24	4.95b		5.89	5.9b	
Cut 8	At level of MSP	5.40	5.60 ab	0.01	4.03	3.80a	0.01	4.55	4.5ª	0.01
	3 mm from MSP	4.80	5.05a		3.23	3.00a		3.83	3.6ª	
	6 mm from MSP	3.88	4.15a		2.62	2.35a		3.10	2.95ª	
Cut 12	At level of MSP	4.62	4.80a	0.01	3.45	3.40a	0.01	3.89	3.95ª	0.01
	3 mm from MSP	3.60	3.90a		2.71	2.50a		3.05	2.95ª	
	6 mm from MSP	2.48	2.45a		1.88	1.60a		2.11	1.7ª	
Cut 16	At level of MSP	3.71	3.70ab	0.01	5.20	4.80a	0.01	3.69	3.7ª	0.01
	3 mm from MSP	3.11	3.10a		5.01	4.75a		2.89	2.95ª	
	6 mm from MSP	1.94	1.60a		5.24	4.95a		1.84	1.5ª	

* Kruskal-Wallis test for lateral thickness comparison, p<0.05 significant* Dunn’s post-hoc test for lateral thickness comparison: equal letters, significant difference, p<0.05

## Data Availability

The datasets used and/or analyzed during the current study are available from the corresponding author.
